# Changes in the thermal stress across Europe between 1940–2023

**DOI:** 10.1007/s00484-025-03043-x

**Published:** 2025-10-07

**Authors:** Luminiţa Mărmureanu, Bogdan Antonescu, Dragoş Ene, Simona Andrei, Raluca Turcu

**Affiliations:** 1 National Institute for Earth Physics, Călugăreni 12, 077125 Măgurele, Ilfov Romania; 2 Faculty of Physics, University of Bucharest, Atomiştilor 405, 077125 Măgurele, Ilfov Romania; 3 Faculty of Forestry, Ştefan cel Mare University, Universităţii 13, 720229 Suceava, Suceava Romania; 4 National Institute of Research and Development for Optoelectronics INOE2000, Atomiştilor 409, 077125 Măgurele, Ilfov Romania

**Keywords:** Universal thermal climate index, Drivers, Climate change, Bioclimatology

## Abstract

Long-term changes (1940–2023) in thermal stress and its drivers in Europe were investigated based on the Universal Thermal Climate Index (UTCI) extracted from the ERA5-HEAT reanalysis. Furthermore, a vulnerability index was introduced to assess the impact of thermal stress. The results reveal a rapid continental shift in thermal stress toward milder conditions. Although cold stress is declining fastest in northern regions (more than 4 hours$\cdot$decade$^{-1}$), heat stress is intensifying across southern Europe (more than 3 hours$\cdot$decade$^{-1}$). Such changes are driven by an increase in 2 m air temperature (between 0.2 up to 0.6$^\circ$C$\cdot$decade$^{-1}$) and mean radiant temperature, particularly over northern and central Europe, and by changes also in relative humidity and wind speed. One of the key findings of this study is the decline in wind speed in specific areas of central and eastern Europe, leading to an increase in UTCI. Monthly analyzes show that winters warm the fastest (e.g., January cold stress decreases by 1.93%$\cdot$decade$^{-1}$), while summer heat stress peaks in July (0.27%$\cdot$decade$^{-1}$). In particular, the late spring months (e.g., May) also exhibit a positive trend, contributing to an extended warm season in Europe. Although European UTCI trends generally show a migration of thermal stress categories toward no to moderate-stress, city-level findings highlight more pronounced effects in continental cities such as Milan, Italy, and less pronounced effects in coastal areas like Madrid, Spain compared to cities of similar latitude. Furthermore, Heat Vulnerability Index reveals that highest vulnerability in southern and eastern Europe. These findings support policymakers in developing science-based measures to mitigate thermal risks, addressing both climatic and socioeconomic vulnerabilities in an era of rapid urbanization and climate change.

**Supplementary Information:**

The online version contains supplementary material available at 10.1007/s00484-025-03043-x.

## Introduction

In recent decades, anthropogenic climate change has led to an increase in the frequency and intensity of extreme high-temperature events worldwide, with heat waves showing an increase in frequency, duration, and intensity (IPCC 2021). The Sixth Assessment Report of the Intergovernmental Panel on Climate Change indicated that these trends will continue throughout the 21st century, even under moderate emission scenarios (IPCC 2021). Such changes pose significant challenges for society, including increased heat-related mortality (e.g. Masselot et al. 2025), impacts on mental health (e.g., Thompson et al. 2023), and agricultural losses, while simultaneously destabilizing ecosystems through species redistribution and biodiversity losses (IPCC 2022). Understanding the spatio-temporal evolution of thermal extremes therefore represents a priority, particularly regarding their human health impacts through direct physiological stress and indirect socioeconomic consequences.

Exposure to extreme temperatures, whether excessively high or low, can significantly affect human health and well-being. Previous research has explored how individuals physiologically respond to thermal stress, their levels of vulnerability, and their capacity to adapt (e.g., Belval and Morrissey 2020; Meade et al. 2020; Folkerts et al. 2020). Although both heat and cold stress pose significant risks, epidemiological studies demonstrate that heat-related mortality currently exceeds cold-related mortality in most mid-latitude regions, with this disparity projected to widen under climate change (e.g., Vicedo-Cabrera et al. 2021; Masselot et al. 2025). This evolving risk landscape requires biometeorological metrics that holistically assess human-environment heat exchange.

To assess thermal stress on human health, well-being, and productivity, a variety of biometeorological indices have been developed (e.g., Błażejczyk et al. 2012; Zare et al. 2018). Among these, the Universal Thermal Climate Index (UTCI) (Błażejczyk 2021), established through COST Action 730 (Jendritzky et al. 2007), offers an equivalent temperature that reflects the physiological responses of humans to fluctuations in the outdoor thermal environment. It does so by incorporating factors such as 2m air temperature, relative humidity, wind speed, and mean radiant temperature (Jendritzky et al. 2012; Bröde et al. 2013). Thus, UTCI can be used as a tool for understanding the impact of climate change on human health and for guiding climate adaptation strategies (e.g., Romaszko et al. 2022). When combined with high-resolution climate data (e.g., Yang et al. 2024), it can also help identify localized hotspots of thermal stress, facilitating more precise planning and intervention.

Given that the UTCI is applicable to a wide range of climatic conditions, recent studies have analyzed the historical changes in the UTCI in various regions, including the Caribbean, South Africa, and Europe. For example, using both the UTCI and the Heat Index, Di Napoli et al. (2020) evaluated thermal stress in the Caribbean region between 1980 and 2019. Their results showed that the most significant increases in heat stress occurred during the rainy season (that is, August–October), with trends indicating an increase exceeding 0.2$^\circ$C$\cdot$decade$^{-1}$ in some areas, reaching up to 0.45$^\circ$C$\cdot$decade$^{-1}$ in regions such as the Lesser Antilles. This increase in heat stress was attributed to rising air temperatures, increased radiation, and decreased wind speeds. These findings underscore the multifaceted nature of thermal stress, driven by large-scale climate patterns and local environmental factors.

Using a similar methodology, Roffe et al. (2023) analyzed the ERA5-HEAT dataset to investigate changes in thermal stress in South Africa from 1979 to 2021. Their findings indicate that southern Africa experiences a wide spectrum of thermal stress categories, ranging from very strong cold stress to extreme heat stress. Statistically significant increases in heat stress were observed in many regions, particularly in spring (i.e, 0.28$^\circ$C$\cdot$decade$^{-1}$) and summer (0.23$^\circ$C$\cdot$decade$^{-1}$), although a few areas showed statistically significant decreases in thermal stress for the same season. In addition, this study highlighted regions in southern Africa that are particularly vulnerable to pronounced changes in thermal stress, emphasizing the necessity of incorporating these findings into decision-making processes for outdoor activities.

In Europe, trends in UTCI have been examined at both the city and the country levels using data from weather stations. Kuchcik et al. (2021) analyzed data from 24 representative weather stations in Poland, spanning 1951 to 2018, with a mean UTCI increase of 0.52$^\circ$C$\cdot$decade$^{-1}$. Their results also indicated a decrease in the number of days with cold stress by 4 days$\cdot$decade$^{-1}$, especially in northeast and eastern Poland and the Carpathian foothills, along with an increase in the number of days with heat stress by 0.8 days$\cdot$decade$^{-1}$, notably in eastern and southeastern Poland. More recently, Błażejczyk and Twardosz (2023) identified similar shifts in both cold and heat stress occurrences using data recorded from 1826 to 2021 at the Jagiellonian University station in Krakow, Poland. For Krakow, the trend in annual mean UTCI values was 0.27$^\circ$C$\cdot$decade$^{-1}$, with the most pronounced changes occurring in winter (0.43$^\circ$C$\cdot$decade$^{-1}$) and the least in summer (0.10$^\circ$C$\cdot$decade$^{-1}$). During the last eight decades, days of cold stress decreased by 1.8 days$\cdot$decade$^{-1}$, whereas heat stress days increased by 0.5 days$\cdot$decade$^{-1}$, exhibiting steeper changes in recent decades. In Serbia, Milošević et al. (2023) evaluated UTCI data from 2000 to 2020 at 27 weather stations, showing an upward trend in annual UTCI (i.e., >2$^\circ$C$\cdot$decade$^{-1}$) at 24 stations, while three stations in central Serbia exhibited decreasing trends. Pantavou et al. (2024) conducted a detailed analysis of Greece’s thermal bioclimate from 1991 to 2020 showing an increase in thermal stress, particularly in the eastern mainland and low-lying regions. Furthermore, two major heatwaves were examined in 2021 and 2023, showing a notable increase in the duration of extreme heat stress, especially in mainland cities, where the urban heat island effect (UHI) amplifies adverse conditions. Other studies have concentrated on broader areas using the ERA5-HEAT dataset, such as Antonescu et al. (2021), who evaluated changes in UTCI between 1979 and 2019 for different regions of Europe based on the Köppen–Geiger climate classification (Köppen 1936; Peel et al. 2007; Beck et al. 2018). The results showed that the annual number of hours with heat stress increased in all analyzed Köppen–Geiger climate sub-classes, most notably in southern Europe. These consistent findings underscore a continent-wide trend towards elevated thermal stress conditions, which requires sustained monitoring and adaptation efforts. Such shifts in thermal stress substantially affect human well-being, including health outcomes (e.g., Ghadam et al. 2021; Urban et al. 2021; Romaszko et al. 2022; Khodadadi et al. 2022; Nastos and Saaroni 2024) and workforce productivity (e.g., Zare et al. 2019). Given the compounding influence of demographic shifts, infrastructure limitations, and socio-economic disparities, these findings underscore the critical need for holistic adaptation measures that integrate urban design, public health strategies, and community engagement.

Despite these findings, knowledge gaps persist regarding Europe’s long-term changes in thermal stress, the diverse atmospheric drivers that influence it, and the trend of different UTCI categories. Moreover, Europe’s climatic heterogeneity and the distinct urban environments across the continent underscore the need for integrated analyses that cover extended temporal scales and capture localized dynamics. Due to the coarse spatial resolution (i.e., 0.25$^{\circ }$) of the ERA5–HEAT UTCI dataset, localized phenomena such as the UHI effect cannot be explicitly resolved. The aim of this paper is therefore to investigate long-term changes in thermal stress in Europe between 1940 and 2023 using UTCI. To achieve this, we first analyze the historical evolution of UTCI and its main drivers, thus examining how 2m air temperature, relative humidity, wind speed, and mean radiant temperature have jointly influenced thermal conditions over more than eight decades. We then explore monthly and regional variations in UTCI, with particular attention to the distribution of UTCI stress categories shifted over time. This approach addresses not only the intensification of extreme conditions, but also the shifts in no to moderate thermal stress that can still be impactful over time. Furthermore, we complement the continental-scale assessment with a focus on 118 European cities from 42 countries, where the compound effects of climate change and urbanization can significantly exacerbate heat stress. Finally, we assessed vulnerability to thermal stress by examining how heat exposure affects at-risk groups, particularly older adults and outdoor workers and by analyzing how these risks intersect with income distribution across Europe. This study aims to inform policymakers, urban planners, and public health officials throughout Europe by providing a historical perspective on changes in thermal stress in the past eight decades.

This paper is organized as follows. Section “Data and methods” describes the data and methodologies used. Section “UTCI and ERA5-HEAT reanalysis” describes the ERA5-HEAT dataset. The methodological approaches for UTCI classification are presented in Section “UTCI classification and trends” and the analyze of UTCI drivers in Section “UTCI drivers”. The changes in thermal stress at city level are detailed in Section “City-level thermal stress analysis”. The data and methodology for developing the Heat vulnerability index is presented in Section “Heat vulnerability index (HVI)”. Section “Spatial and temporal distribution of UTCI over Europe” presents the spatial and temporal distribution of UTCI in Europe between 1940 and 2023. The long-term changes in the drivers of UTCI are analyzed in Section “Climatology and trends in the UTCI drivers”. Annual, monthly, and spatial changes in no to moderate, cold, and heat stress categories over Europe are shown in Sections “Annual changes in UTCI”–“Spatial distribution of thermal stress classes”. Section “Changes in thermal stress classes for European cities” details the changes in UTCI at city level, and Section “Vulnerability to heat stress” details about vulnerability to heat stress. Section “Discussion and recommendations” discusses the findings in terms of public health implications and adaptation strategies. Finally, Section “Conclusions” concludes with a summary of key findings.

## Data and methods

### UTCI and ERA5-HEAT reanalysis

**Fig. 1:**
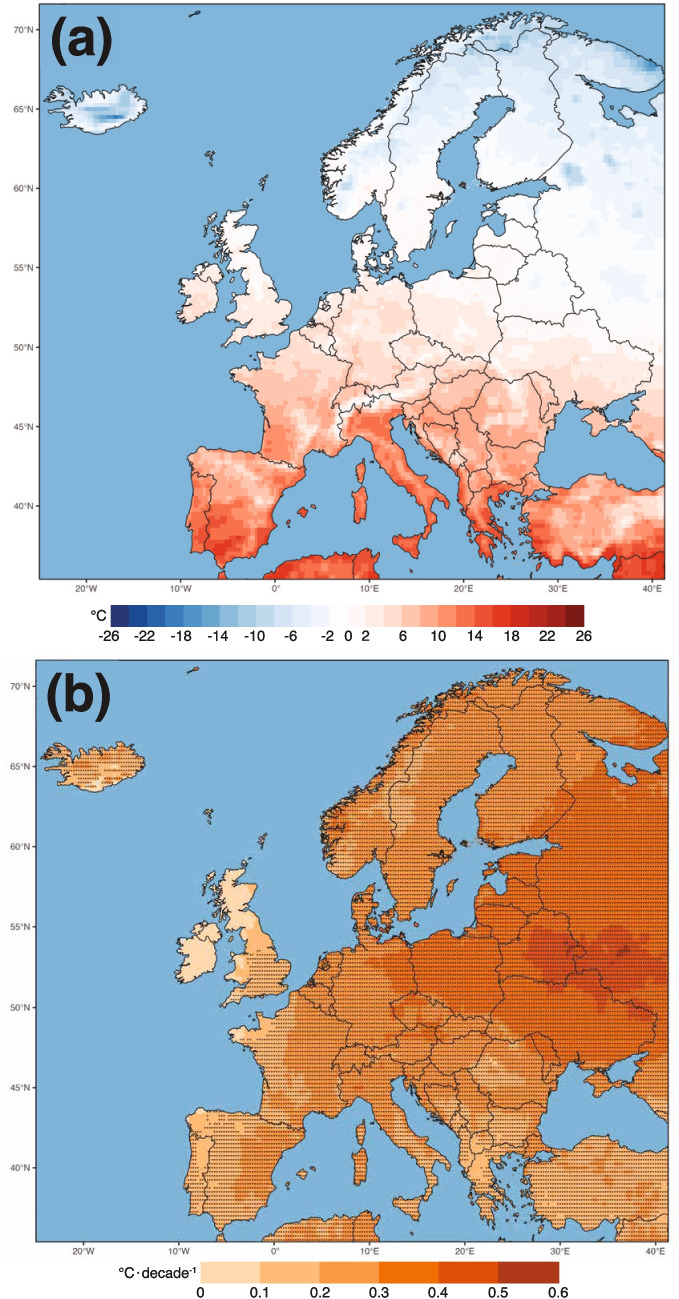
(a) Mean UTCI 1940–2023 ($^\circ$C). The corresponding UTCI stress category can be found in Table S1. (b) Trends in UTCI over Europe ($^\circ$C$\cdot$decade$^{-1}$) represented by Sen’s slope coefficients. Black dots are indicating statistically significant trends based on Mann-Kendall test (*p* < 0.05)

The primary dataset used in this study is the ERA5-HEAT reanalysis (Di Napoli et al. 2020), provided by the Copernicus Climate Change Service (C3S) (Di Napoli et al. 2024). ERA5-HEAT builds on the fifth generation global atmospheric reanalysis produced by the European Center for Medium-Range Weather Forecasts (ECMWF), known as ERA5 (Hersbach et al. 2020). The global output (except Antarctica) has a temporal resolution of 1 hour on a 0.25$^{\circ }$ spatial grid. Over the European domain, this corresponds to a uniform north–south spacing of approximately 27.8 km, while the east–west spacing varies from about 23.0 km at 34.3$^{\circ }$N to around 9.0 km at 71$^{\circ }$N, averaging to approximately 17 km at mid-latitudes. In this article, we analyzed only the values for the European domain defined as 34.3$^{\circ }$N–71$^{\circ }$N and 24$^{\circ }$W–41.5$^{\circ }$E, including parts of western Russia up to 41.5$^{\circ }$E (Fig. 1). The study period spans from 1940 to 2023, providing a comprehensive perspective on the trends in thermal stress throughout Europe.

UTCI, expressed in equivalent temperature ($^\circ$C), represents the air temperature of a reference environment that would produce the same physiological stress on a human body as the actual environmental conditions, based on metrics such as core temperature regulation and sweat rate. In the reference environment, the 2m air temperature (T2M) was equal to the mean radiant temperature (MRT), the relative humidity (RH) was set at 50%, and the wind speed measured at 10 m height (WS) at 0.5 m $\cdot$ s $^{-1}$ (representing calm air) (Jendritzky et al. 2012). UTCI values are calculated using a sixth-order polynomial regression equation, using T2M, RH, WS, and MRT as input parameters, as well as an adaptive clothing model that adjusts based on environmental conditions (Havenith et al. 2012). Based on the calculated UTCI values, the thermal comfort levels are classified into 10 different categories of thermal stress, each representing a specific level of physiological stress from extreme cold to extreme heat (Table S1). These categories facilitate a standardized assessment of thermal comfort and stress in diverse climates and time periods.

### UTCI classification and trends

The ten UTCI categories were grouped in this article in three thermal stress classes: (1) no to moderate thermal stress when -13$^{\circ }$C $\le$ UTCI $\le$ 32$^{\circ }$C (i.e., UTCI categories “moderate heat stress”, “no thermal stress”, “slight cold stress”, and “moderate cold stress”; Table S1), (2) cold stress when UTCI < -13$^{\circ }$C (i.e., UTCI categories “strong cold stress”, “very strong cold stress”, and “extreme cold stress”), and (3) heat stress when UTCI >32$^{\circ }$C (i.e., UTCI categories “strong heat stress”, “very strong heat stress”, and “extreme heat stress”). For each grid cell, the number of hours in each thermal stress class was extracted for analysis.

Temporal trends in UTCI and the three UTCI classes were calculated using the nonparametric Mann-Kendall trend test and Sen’s slope estimator (Kendall 1975; Sen 1963). A trend was considered statistical significant using *p* value < 0.05. This analysis was implemented using the *rtrend* R package^1^.

### UTCI drivers

ERA5 reanalysis single levels hourly dataset was used C3S (2023) to study the changes in the drivers of UTCI. Specifically, we extracted 2m air temperature, 2m dewpoint temperature, and the 10m u and v components of the wind from the ERA5 reanalysis. Subsequently, the relative humidity was calculated using the 2m temperature and dewpoint, following the approach outlined in Lawrence (2005) (equation 8). The 2m air temperature values for the UTCI calculations were restricted to the range of -50$^\circ$C to +50$^\circ$C (Bröde et al. 2012). Based on the guidelines of Froehlich and Matzarakis (2015), the wind speed used in the UTCI calculations should be between 0.5–17 m$\cdot$s$^{-1}$. Finally, MRT was extracted from the ERA5–HEAT dataset. Additionally, the UTCI algorithm constrains the difference between MRT and T2M, as model inputs, to a range of -30$^{\circ }$C to +70$^{\circ }$C (Di Napoli et al. 2021).

To isolate the individual influence of each meteorological variable on UTCI, a “one-at-a-time” perturbation approach was used. First, the mean fields of the four inputs required for UTCI — T2M, RH, WS, MRT — were calculated over two distinct periods: (i) a historical period (i.e., 1940–1960) denoted as: $\left( T2M_0, RH_0, WS_0, MRT_0\right)$, and (ii) a recent period (i.e., 2003–2023) denoted as: $\left( T2M_1, RH_1, WS_1, MRT_1\right)$. Second, UTCI was calculated for historical: $UTCI_0\left( T2M_0, RH_0, WS_0, MRT_0\right)$ and recent period: $UTCI_1\left( T2M_1, RH_1, WS_1, MRT_1\right)$ for the entire domain and for each of the 21 years of both periods. To compute UTCI, the R package *ClimInd* : *ClimateIndices*^2^ was used. Third, to quantify how each variable separately impacted UTCI, a single meteorological variable from the recent fields was substituted into the historical dataset while keeping the other three variables at their historical values. This resulted in four additional UTCI fields:

$$\begin{aligned} UTCI_{T2M} = UTCI\left( T2M_1, RH_0, WS_0, MRT_0\right) \\ UTCI_{RH} = UTCI\left( T2M_0, RH_1, WS_0, MRT_0\right) \\ UTCI_{WS} = UTCI\left( T2M_0, RH_0, WS_1, MRT_0\right) \\ UTCI_{MRT} = UTCI\left( T2M_1, RH_0, WS_0, MRT_1\right) \end{aligned}$$

Fourth, for each driver $X \in \{T2M, RH, WS, MRT\}$ the change in UTCI due to the change in that single variable from the historical to the recent period was calculated as $\Delta \textrm{UTCI}_X = \textrm{UTCI}_X - \textrm{UTCI}_0$. Thus, $\Delta \textrm{UTCI}_{T2M}$ captures the effect of changing temperature, and likewise for the other three variables for the entire domain and each of the 21 years of the historical period. The resulting maps show the average effect of each variable. To account for the interaction between the inputs for UTCI, a “interaction” term was calculated:

$$\begin{aligned} \Delta \textrm{UTCI}_{interaction}&= \Delta \textrm{UTCI}_{all} - (\Delta \textrm{UTCI}_{T2M} + \Delta \textrm{UTCI}_{RH} \\&+ \Delta \textrm{UTCI}_{WS} + \Delta \textrm{UTCI}_{MRT}) \end{aligned}$$

where $\Delta \textrm{UTCI}_{all} = UTCI_1 - UTCI_0$. Thus, if the interaction term is positive, then all input variables change together (that is, the UTCI is greater than the sum of separate changes). If the interaction term is negative, cancellation or damping effects occur among the drivers of UTCI.

Using only two time periods (i.e., 1940–1960 and 2003–2023) provides an information of which variable has most influenced the long-term change in mean thermal stress, but offers limited insight into seasonal or annual fluctuations. Furthermore, this approach can obscure intermediate changes, gradual progressions, or non-linear trends that may have occurred between the two averaging periods. Despite these limitations, the approach can provide valuable details because the goal is to identify the dominant drivers from a long-term perspective.

### City-level thermal stress analysis

To assess changes in thermal stress on the urban scale, we selected a representative selection of cities across Europe. For each country, our selection criteria encompassed three key urban typologies: (i) the capital city, (ii) the second largest city by population, and (iii) the principal tourist destination (see Fig. S1). This approach was designed to capture the diversity of urban environments and their unique climatic challenges. In total, 118 cities from 42 European countries were included in the analysis (see Table S2). For microstates such as Liechtenstein, Monaco, San Marino, and Vatican City, only the capital was analyzed due to their limited geographical extent. For the category of tourist cities, we prioritized cities based on metrics such as annual visitor numbers and inclusion among UNESCO-listed sites. For each selected city, the analysis used the grid point in the ERA5–HEAT dataset that was close to the geographic center of the city.

### Heat vulnerability index (HVI)

To assessed the vulnerability to heat stress in Europe we used socio-economic data from EUROSTAT, the statistical office of the European Union^3^. Vulnerability (V) was defined (e.g., Szagri et al. 2023) as a function of exposure (E), sensitivity (S), and adaptive capacity (AC):

$$\text {{\textbf {V}}ulnerability} = \text {{\textbf {E}}xposure} + \text {{\textbf {S}}ensitivity} - \text {{\textbf {A}}daptive {\textbf {C}}apacity}$$

For exposure, we considered the mean annual number of hours with heat stress (*H*). For sensitivity we extracted from EUROSTAT three indicators: (1) the percentage of population age 65 and older ($P_{65+}$), (2) the percentage of population at risk of poverty and social exclusion ($P_{poverty}$), and (3) percentage of people age 15–74 employed in agriculture, forestry, fishing and construction (i.e., outdoor workers, $P_{outdoor}$). The gross domestic product (GDP) at current market prices (in Euro per inhabitant, *GDP*) was used as an indicator for the adaptive capacity. All the data were extract at NUTS2 (Nomenclature of Territorial Units for Statistics) level, which are regions that are large enough to capture meaningful economic and demographic patterns. These socio-economic indicators were then combined into a simple HVI using mean values between 2019–2023 for each NUTS2.

Different methods for making HVIs yield varying results due to choices in indicators, scale, normalization, and weighting (Niu et al. 2021; Szagri et al. 2023). Our study emphasizes data availability and comparability across European regions (NUTS-2), using equal variable weighting and omitting, for example, urban factors. This approach supports broad assessments but may miss localized vulnerabilities. The results should be viewed with these limitations, and future research could incorporate more detailed, location-specific data. First, all variables were normalized using the min-max normalization:

$$P' = \frac{P - P_{\text {min}}}{P_{\text {max}} - P_{\text {min}}}$$

where $P \in \{H, P_{65+}, P_{\text {poverty}}, P_{\text {outdoor}}\}$. For adaptive capacity indicators, higher values imply lower vulnerability; therefore, the values is:

$$GDP' = 1 - \frac{GDP - GDP_{\text {min}}}{GDP_{\text {max}} - GDP_{\text {min}}}$$

Second, the HVI is computed as the arithmetic mean of the normalized variables indicators:

$$\textrm{HVI} = \frac{1}{n} \sum _{i=1}^{n} X'_i$$

where $X'$ represent the four socio-economic indicators and exposure. The computed HVI offers a harmonized and spatially comparable metric of heat vulnerability, quantifying each NUTS-2 region in Europe’s susceptibility to heat stress on a continuous scale ranging from 0 (very low vulnerability) to 1 (very high vulnerability).

## Results

### Spatial and temporal distribution of UTCI over Europe

The mean UTCI values in Europe between 1940 and 2023 show latitudinal gradients, with southern Europe showing higher mean UTCI values (i.e., >9$^\circ$C), suggesting on average no thermal stress conditions (Table S1), while northern and high-altitude regions provide lower values (i.e., <0$^\circ$C), indicating slightly to moderate and even strong cold stress (Fig. 1a). There is a statistically significant trend in UTCI throughout Europe, with regions in western Europe (e.g., the United Kingdom, Ireland, France, Portugal) and Greece where the trend is not statistically significant (Fig. 1b). There is an area with notable trends in UTCI (>0.3$^\circ$C$\cdot$decade$^{-1}$) stretching over Poland, Lithuania, Latvia, Estonia, Belarus, Ukraine, Moldova, and western Russia. These increasing trends correspond with overarching climate change patterns that result in changes from cold stress to moderate or no stress conditions, for example, in eastern Europe, and toward more heat stress conditions in southern Europe. In contrast, regions without statistical significance may indicate localized climatic stability or variability affected by parameters such as large body water moderation. To better understand these changes in the UTCI, the climatology and trends in the UTCI drivers are presented in the next section.

### Climatology and trends in the UTCI drivers

The mean annual values of the UTCI drivers and their trends are shown in Fig. S2 for 2m air temperature (T2M) and relative humidity (RH) and in Fig. S3 for wind speed (WS) and mean radiant temperature (MRT). There is a strong latitudinal gradient with values ranging below 0$^\circ$C over northern Europe (but also over high-elevation areas, such as the Alps) to above 20$^\circ$C over southern Europe and part of the Balkans (Fig. S2a). Also, there is an increasing and statistically significant trend in temperature throughout the continent, ranging from 0.2–0.6 $^\circ$C$\cdot$decade$^{-1}$ (Fig. S2c). The highest trends are observed over Iceland, Norway, and Sweden, consistent with amplified warming at high latitudes as shown in previous research (e.g., Rantanen et al. 2022). Other regions with relatively high trends (up to 0.4 $^\circ$C$\cdot$decade$^{-1}$) are eastern Spain, northern Italy, and northeastern Europe.

There is a broad pattern of higher RH in northern and central Europe (i.e., 75–80%), and with lower values (i.e., <60%) observed in the Mediterranean basin, reflecting both latitudinal climate differences and the presence of large bodies of water (Fig. S2b). There are several regions characterized by a strong decreasing statistically significant trend (i.e., >-0.8% $\cdot$decade$^{-1}$) in RH such as: Iceland, Norway, northwestern Sweden, northern Spain, northern Italy, and eastern Turkey (Fig. S1d). As shown in Fig. S2c, these are likely temperature-driven drying trends. Increasing trends (i.e., >0.2% $\cdot$decade$^{-1}$) in RH are observed over Ireland and Hungary, southern Germany and parts of Finland. These may reflect enhanced moisture transport or local cooling effects (e.g., greater precipitation and evapotranspiration feedback).

Figure S3a shows that higher wind conditions (i.e., >4 m$\cdot$s$^{-1}$) in northwestern Europe (i.e., the United Kingdom, North Sea, North Atlantic regions) and lower winds (i.e., <1.5 m$\cdot$s$^{-1}$) in continental interiors and along the northern Mediterranean basins (Fig. S3a). Several regions in northern and northeastern Europe show increasing statistically significant trends over Iceland, Norway, Sweden and northwestern France, and decreasing over Hungary, southern Romania, and parts of eastern Europe (Fig. S3c). However, wind data often carries larger uncertainties, so small changes may be less robust. Mean radiant temperature displays a strong north-to-south gradient with values below 10$^\circ$C over far northern Europe and mountainous areas to values above 25$^\circ$C over southern Europe (Fig. S3b). Nearly all of Europe shows a pronounced increasing statistically significant trend in MRT (i.e., >0.2 $^\circ$C$\cdot$decade$^{-1}$), with the exception of the Balkan region (Fig. S3d). The changes in MRT align well with the warming pattern of the parameters for its calculations (e.g., surface albedo, cloud cover, and overall radiative forcing).

**Fig. 2:**
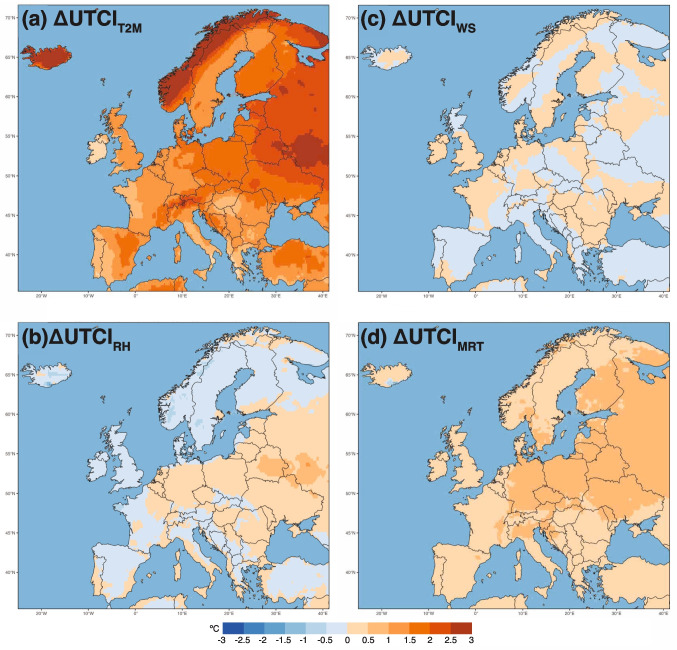
The spatial distribution of (a) $\Delta$ $\textrm{UTCI}_{T2M}$, (b) $\Delta$ $\textrm{UTCI}_{RH}$, $\Delta$ $\textrm{UTCI}_{WS}$ and $\Delta$ $\textrm{UTCI}_{MRT}$. See Section “UTCI drivers” for details on how these terms are defined in “one-at-a-time” perturbation approach

Throughout much of Europe, substituting the recent (2003–2023) T2M field into the calculation of UTCI for the historical period (1940–1960) results in a high positive value for $\Delta UTCI_{T2M}$ (i.e. >2 $^\circ$C) in particular over northern and eastern Europe (Fig. 2a). The higher T2M alone thus substantially increases the value of UTCI in these regions. This temperature-driven increase in UTCI is also observed in parts of southern Europe, such as northeastern Spain. Substituting RH from 2003–2023 can reduce UTCI in some areas, for example, Iceland, Scandinavia, the United Kingdom, and large areas of Spain and France (Fig. 2b). In contrast, parts of central and eastern Europe exhibit an increase in UTCI. Many regions in central and eastern Europe show positive values for $\Delta UTCI_{WS}$, implying declines in wind speed which are increasing further UTCI (Fig. 2c). Compared with $\Delta UTCI_{T2M}$, the values $\Delta UTCI_{WS}$ are typically lower, but they can still be important at regional scales. Finally, $\Delta UTCI_{MRT}$ show positive values across the entire continent (Fig. 2d). Thus, T2M often emerges as the dominant driver in the changes of UTCI from the 1940–1960 baseline to the recent period (2003–2023), with MRT also having a contribution. RH and WS effects are more uneven across the continent.

The interaction term is mainly negative across Europe, with the highest values over Norway and northeastern Sweden (i.e., >-0.08 $^\circ$C) (Fig. S4). This indicates that some compensation and dampening among the four variables (i.e., one variable’s effect is cancelling another variable’s effect when all the variables are considered compared to summing them individually). Over northern Europe, the United Kingdom, and northwestern France, the interaction term shows positive values indicating that simultaneous changes in T2M, RH, WS, and MRT result in a higher UTCI difference than the sum of their individual contributions. Overall, the magnitude of $\Delta UTCI_{interaction}$ indicates that while T2M and MRT are the main drivers of the changes in UTCI, nonlinearities in the UTCI formula can locally lead to an increase or decrease of the thermal stress.

This shows that in highly populated urban areas, adaptation measures in response to UTCI variations should consider wind speed patterns, relative humidity, and mean radiant temperature to guide urban planning decisions. Strategies may include optimizing natural ventilation, implementing targeted green infrastructure to regulate humidity, and designing street orientations that account for seasonal variations in wind direction and moisture levels.

### Annual changes in UTCI

**Fig. 3:**
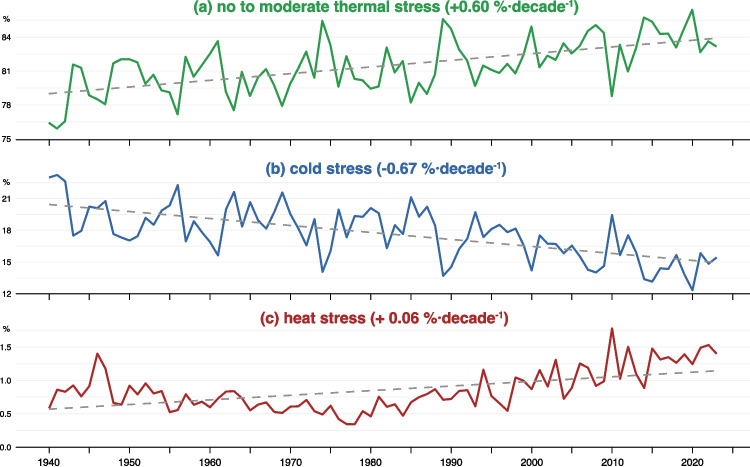
Annual percentage of hours in (a) no to moderate thermal stress class (i.e., -13$^\circ$C < UTCI < 32$^\circ$C, Table S1) (green line), (b) cold stress class (i.e., UTCI $\le$-13$^\circ$C) (black line), and heat stress class (i.e., UTCI $\ge$ 32$^\circ$C) (red line) from 1940 to 2023 across Europe. The dashed gray lines show the Sen’s slope accompanied by the Mann–Kendall test results. All three trends are statistically significant (p < 0.05)

The annual changes in the three aggregated categories of thermal stress as a percentage of the total annual hours with thermal stress are shown in Fig. 3. The percentage of hours in the categories of no to moderate and heat stress categories exhibited an increase of +0.6%$\cdot$decade$^{-1}$, and +0.06%$\cdot$decade$^{-1}$, respectively. At the same time, the cold stress category shows a pronounced, highly significant decreasing trend (i.e., -0.67%$\cdot$decade$^{-1}$), indicating a reduction in extreme cold conditions during the entire study period. These changes in heat stress categories are consistent with observed increases in average summer temperatures and more frequent heatwaves (e.g., Ionita et al. 2024). Thus, while conditions associated with the cold category are decreasing most rapidly and those associated with the heat category are increasing from a lower baseline, the net effect is a progressive shift toward milder thermal conditions (i.e.,the categoryy of no to moderate stress) overall.

### Monthly changes in UTCI

**Fig. 4:**
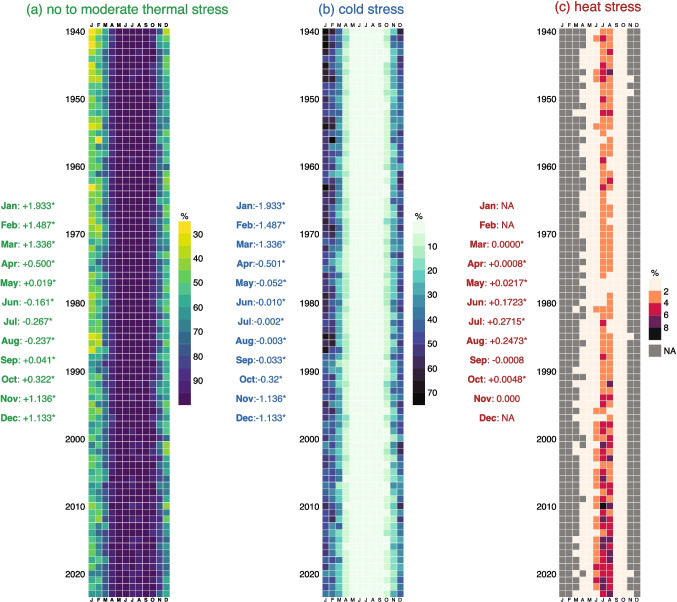
Monthly percentage of hours in three aggregated UTCI classes: (a) no to moderate thermal stress, (b) cold stress, and (c) heat stress—shown on a year-month grid from 1940 to 2023. The cells represent the fraction of hours (in %) for each category within each month and year (see color bar scales). Gray cells in the heat stress panel represents that there are no values for a specific month and year. Along the left side of each panel, the text denotes the Sen’s slope (with * indicating p < 0.05) for each month

The monthly distribution of the percentage of hours in the category of no to moderate thermal stress shows high values (i.e., >85%) from April to October, a pattern that is consistent throughout the study period (Fig. 4a). There is an increasing statistically significant trend in the percentage of hours in this category for every month with the exception of June–August, which is characterized by a decreasing trend with a maximum in July (i.e., -0.267%$\cdot$decade$^{-1}$). The highest trend is in January (i.e., +1.993%$\cdot$decade$^{-1}$) and is followed by February (i.e., +1.487%$\cdot$decade$^{-1}$). This is associated with changes in the monthly percentage of hours within the cold stress category (Fig. 4b). Again, January (i.e., -1.993%$\cdot$decade$^{-1}$) and February (i.e., -1.487%$\cdot$decade$^{-1}$) show the highest decreasing trend for cold stress, representing a near-perfect shift from cold stress to no to moderate thermal stress in these months. Meanwhile, changes in the monthly percentage of heat stress show a modest but increasing trend in late spring and summer (that is, May–August) with the highest increase observed in July (i.e., +0.271%$\cdot$decade$^{-1}$) (Fig. 4c). Together, this monthly perspective underscores a clear long-term trend with winters (December–February) becoming milder, with a broad area of the year now exhibiting more moderate conditions, and late spring and summer months gradually experiencing higher incidences of heat stress.

### Spatial distribution of thermal stress classes

**Fig. 5:**
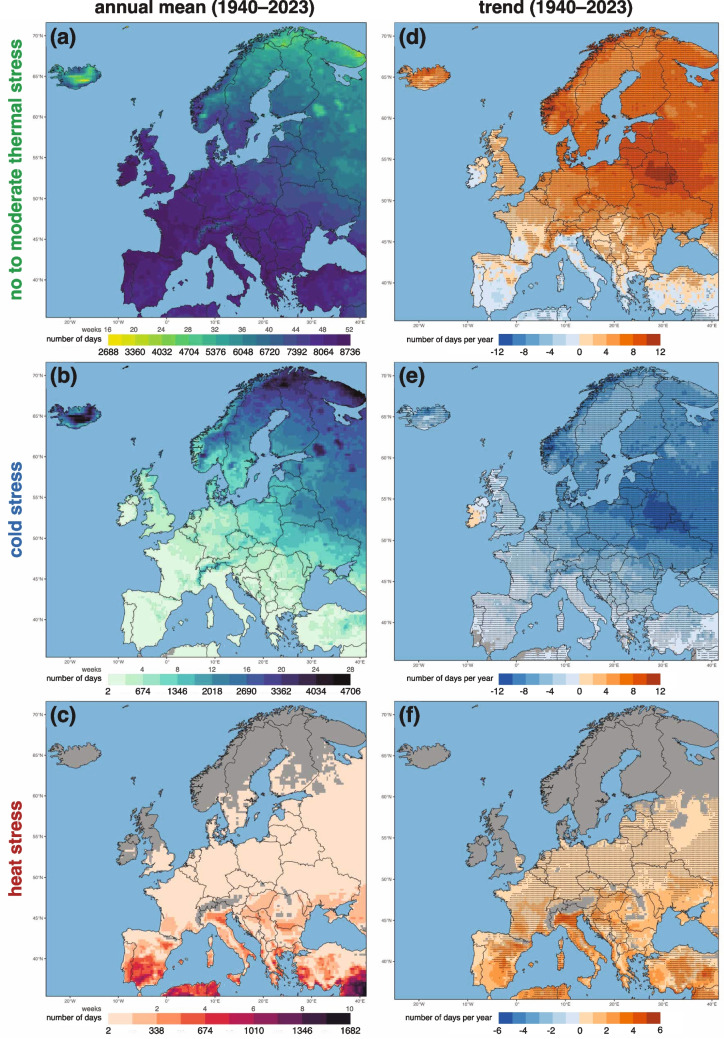
Spatial distribution of the three aggregated categories showing (a)–(c) the annual mean and (d)–(f) trend (right column) between 1940–2023. Dots in the panel (d)–(f) are indicating statistically significant monotonic trends (p < 0.05). For panel (a)–(c) the colorbars are also annotated to indicate the equivalent of the number of days in week. Gray cells in the heat stress panel (c) indicates no hours for the specific class

The spatial distributions of annual mean hours and their long-term trends reveal clear geographic contrasts in Europe’s thermal stress regimes (Fig. 5). Regions of no thermal to moderate stress dominate western, central, southern, and southeastern Europe, where mean annual values commonly exceed 7,300 hours (equivalent to more than 44 weeks per year; Fig. 5a). In contrast, cold stress is most prevalent in northern and northeastern Europe, exceeding 3,000 hours (18 weeks) per year (Fig. 5b). Meanwhile, heat stress is concentrated in the Mediterranean region, where it often exceeds 670 hours (4 weeks), diminishing northward and eastward into central and southeastern Europe (Fig. 5c).

Trend analyses over the 1940–2023 period indicate significant shifts in all three categories. A widespread decline in cold stress days (Fig. 5d) is matched by the corresponding increases in both no to moderate thermal stress (Fig. 5e) and heat stress (Fig. 5f). Eastern Europe exhibits one of the largest decreases in cold stress (on the order of 8–12 hours$\cdot$year$^{-1}$), and an equivalent increase in mild to moderate thermal stress. Northern and central Europe also experience reductions in cold stress, although at slightly lower rates (4–8 hours year$^{-1}$) with an accompanying rise in moderate thermal conditions. In southern Europe, by contrast, a decline in no to moderate thermal stress is overlapped by a growth in heat stress (4–6 hours$\cdot$year$^{-1}$), particularly around the Mediterranean basin.

Collectively, these trends show a redistribution of thermal stress regimes in accordance with a warmer climate, characterized by milder winters (as evidenced by the reduced cold stress) and more frequent or prolonged heat events (reflected in the growing heat stress). The amplification of heat stress in southern Europe underscores the vulnerability of Mediterranean regions to a more warming conditions, with potential implications for human health. Taken together, the results highlight how rising temperatures and decreasing winter severity are driving a shift in Europe’s thermal environment, which is likely to intensify if warming continues along the present trajectories (IPCC 2022).

### Changes in thermal stress classes for European cities

**Fig. 6:**
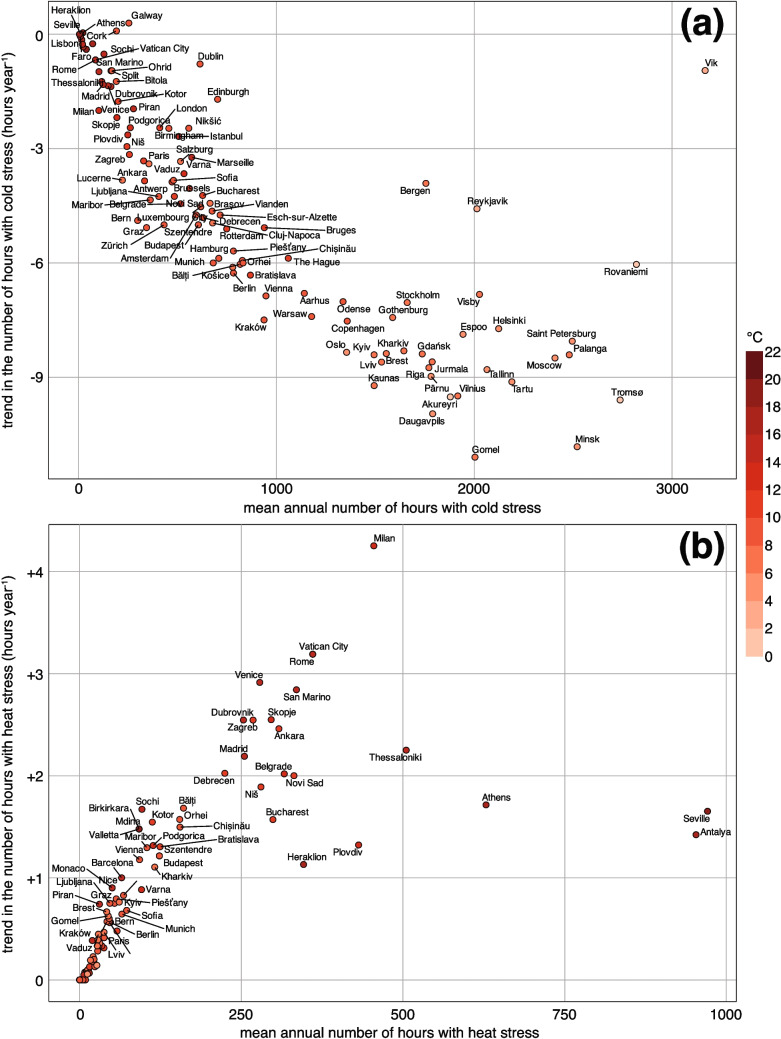
The distribution of 118 European cities based on the mean annual number of hours and trend in the number of hours for (a) cold stress and (b) heat stress classes between 1940–2023. Each city is shaded based on the corresponding mean annual temperature. The details for each city are provided in Table S2

Figure 6 presents a city-level view on how the cold stress and heat stress have evolved during the 1940–2023 period for 118 cities in 42 European countries. Each city is plotted according to its mean annual hours under the respective stress category, while the vertical axis indicates whether these conditions are becoming more frequent (positive trend) or less frequent (negative trend). For the cold stress class, the majority of cities lie in the negative trend area (with the exception of Cork and Galway, Ireland), reflecting a systematic decline in the number of hours of cold stress over the last eight decades (Fig. 6a). Even cities with relatively high baseline cold stress durations, such as Reykjavik and Vik (Iceland) or Rovaniemi (Finland), show significant negative trends of up to -10 hours$\cdot$year$^{-1}$. This pattern indicates that, although these northern-latitude locations still experience the largest absolute number of cold stress hours (that is, more than 2,000 hours annually), they are nevertheless undergoing a reduction in winter severity. Mid-latitude cities, such as Kraków (Poland), Prague (Czech Republic), or Vienna (Austria), show a lower mean annual cold stress (that is, 500–1,500 hours$\cdot$year$^{-1}$) but also show a consistent decreasing trend of -8 to -5 hours$\cdot$year$^{-1}$. This collective decrease across climatically diverse regions points to broad continental warming trends that decrease the duration of cold conditions, likely due to both rising average winter temperatures and shorter cold-season durations.

A different spatial distribution is represented by the heat stress (Fig. 6b). Cities in the Mediterranean and southeastern Europe, including Antalya (Turkey), Seville (Spain), and Athens (Greece) indicate both a high mean annual number of heat-stress hours (often exceeding 750–1,000 hours$\cdot$year$^{-1}$) and strong positive trends (as high as +3 to +4 hours$\cdot$year$^{-1}$). These findings are consistent with increasingly prolonged hot spells, especially around the Mediterranean basin. Central European cities, such as Budapest (Hungary) or Bratislava (Slovakia), occupy the lower left region with fewer heat stress hours (under 200–300 hours annually), though many still exhibit modest positive trends, illustrating a gradual expansion of the summer heat period. Northern European cities, where heat stress hours were historically minimal, often lie near the lower left corner (close to zero annual hours), but in some cases, they, too, show an upward trend, although small. This suggests a northward extension of conditions once largely confined to southern latitudes.

Furthermore, these trends likely reflect not only large-scale climatic shifts but also local factors such as the urban heat island effect, latitude, proximity to oceans or seas, and topographic influences. For example, Copenhagen (Denmark) and Oslo (Norway), both coastal northern cities, show a noticeable reduction in cold stress reflecting warmer winters. This may be explained in part by the moderating influence of maritime conditions, which buffer extreme cold. Barcelona (Spain) and Venice (Italy), while prone to summer heat, exhibit somewhat lower total heat stress hours than inland Mediterranean cities of similar latitude (see, e.g., Madrid, Spain). This indicates the cooling effect of coastal breezes and water-mediated heat capacity. In contrast, cities with dense urban structures and limited green space, such as Milan (Italy), experience amplified summertime heat stress, consistent with localized warming that affects human thermal comfort.

### Vulnerability to heat stress

**Fig. 7:**
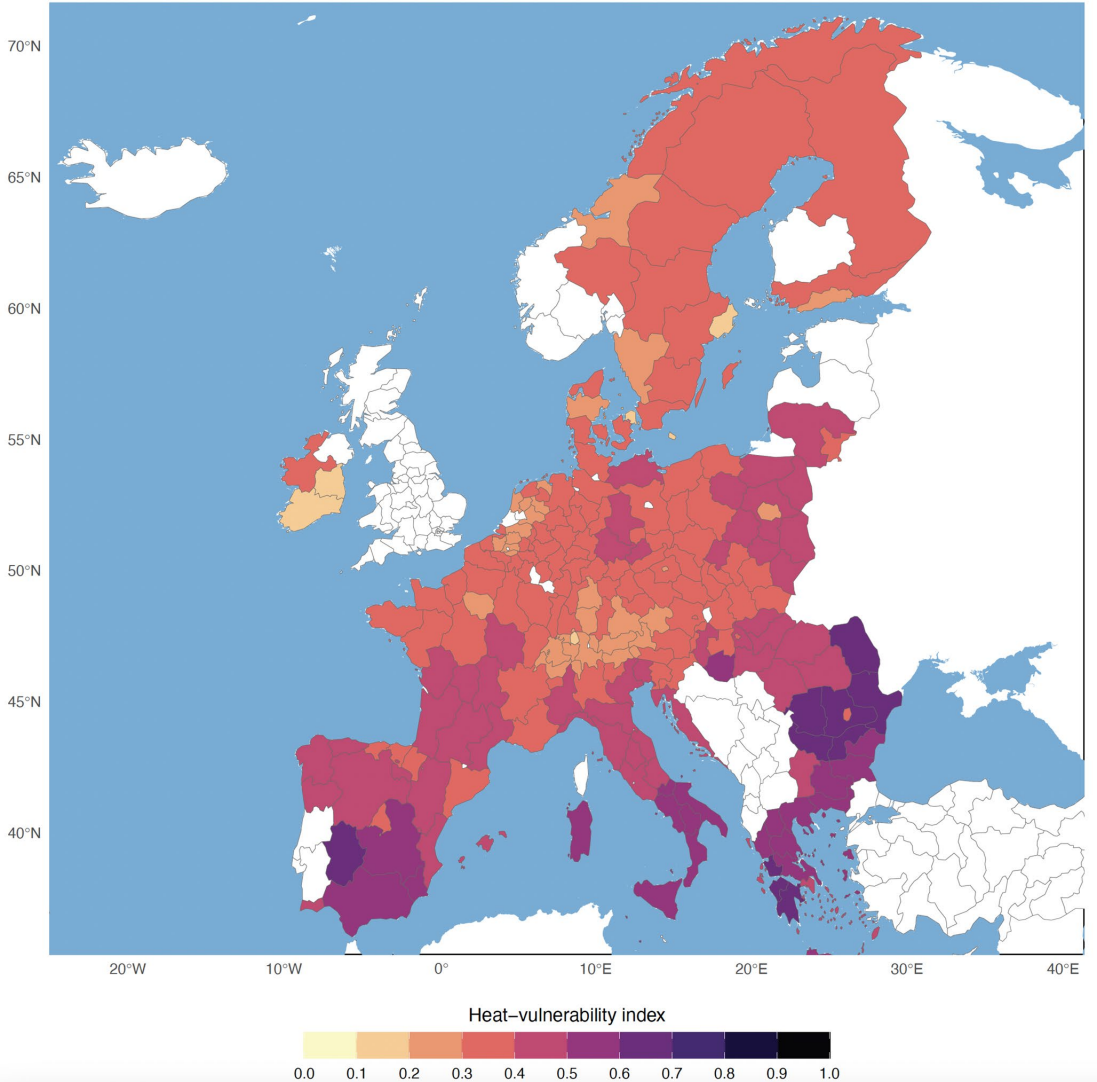
The spatial distribution of the HVI across European NUTS2 regions. The regions shown in white are those for which the socio-economical indicators where not available for the period 2019–2023

The spatial distribution of the Heat Vulnerability Index (HVI) across European NUTS2 regions highlights the geographic disparities in vulnerability to heat exposure (Fig. 7). Regions in southern and eastern Europe, especially in southern Spain, southern Italy, Greece, Bulgaria, and Romania, show high HVI values (i.e., exceeding 0.5). In these areas, a high number of hours with heat stress combined with large elderly populations and high rates of poverty or social exclusion drive up overall vulnerability. The very highest scores (i.e., HVI >0.6) appear in southern and eastern Romania, northwestern Bulgaria, southern Greece, and western Spain. For example, northeastern Romania’s high HVI reflects a pronounced poverty risk and a large share of outdoor workers, despite a relatively moderate number of heat-stress hours. By contrast, southwestern Spain shows similarly high vulnerability driven primarily by a large number of days with heat stress, even though its share of agricultural workers is lower. Most of western and northern Europe displays much lower vulnerability (i.e., HVI < 0.3), where a lower number of hours with heat stress coincide with stronger adaptive capacity, higher GDP per capita. Similarly, major metropolitan areas such as Paris, Warsaw, Madrid and Bucharest also fall into this lower-risk category, likely due to their relatively favourable socio-economic conditions.

## Discussion and recommendations

The European thermal landscape is transitioning to a “new normal”, marked by asymmetric risks. This study indicated how thermal stress shifted across Europe between 1940 and 2023. These changes significantly affect human perception of thermal stress, public health, economic activities, and energy demands. Southern European cities, such as Seville and Antalya, exhibit heat stress trends of +3 to +4 hours$\cdot$year$^{-1}$ (Fig. 6) an effect that was recently observed in the increased mortality during Mediterranean heat waves (e.g., Pantavou et al. 2024). The human population is becoming increasingly exposed to thermal stress as global temperatures rise, with significant variations in thermal tolerance influenced by regional acclimatization, physiological adaptation, and behavioral responses to climate change (e.g., Mastrucci et al. 2022; Meidenbauer et al. 2024). A recent study conducted in the Netherlands over a 23-year period (1995–2017) suggested incremental acclimatization to heat stress as indicated by rising minimum mortality temperatures (Folkerts et al. 2020). Furthermore, by 2050, 41.8–58.6% of the population will experience unfamiliar conditions, 28.5–41.5% uncommon, and 2.1–8.9% unknown conditions, with significant impacts in regions like Spain, the Alps, and the Mediterranean (Dosio et al. 2025).

Outdoor workers, particularly in regions exceeding 250 annual heat stress hours (e.g., Bucharest, Roma, Athena, Fig. 6), face increased risks of productivity loss and heat-related illness (Habibi et al. 2024). Also, the agricultural sector in Mediterranean basins is expected to be further strained by water scarcity and pest proliferation, threatening crop viability (Nelson et al. 2024). Meanwhile, milder winters in northern Europe (Fig. 5b,e) can reduce cold-related morbidity but could destabilize ecosystems adapted to prolonged frost periods.

Shifting thermal regimes also have an impact on energy demands. Northern cities like Reykjavik and Rovaniemi show marked reductions in cold stress (-10 hours$\cdot$year$^{-1}$, Fig. 6), reducing heating needs. In contrast, the cooling demand of southern Europe is rising (Ciancio et al. 2020). For example, Milan’s dense urban area is correlated with amplified heat stress, underscoring the need for climate-responsive actions (e.g., building designs and decentralized renewable energy systems).

Alongside these findings, the urban population in Europe, which reached 75.7% in 2023, has shown a consistent upward trend in recent decades (Eurostat 2024). The doubling of heat stress hours in 2023 compared to 1940 (Fig. 3), combined with the exacerbating effects of the UHI and increased urban population, will considerably affect public health and wellbeing. These can lead to health issues, especially for vulnerable populations such as the elderly and those with pre-existing medical conditions.

Taken together, these demographic and climatic factors highlight the need for integrative vulnerability assessments that consider both thermal exposure and socio-economic context. The HVI used in this study indicates pronounced vulnerability in southern and eastern Europe, reflecting the overlap of intense heat stress exposure and socio-economic factors. This aligns with previous research identifying these regions as climate-health hotspots (Bednar-Friedl et al. 2022), reinforcing the importance of prioritizing resources and developing adaptation strategies that integrate exposure metrics with socio-demographic resilience.

In light of these findings, a range of policy measures is recommended to address the challenges of shifting thermal stress in Europe. Urban adaptation should prioritize green infrastructure (e.g., Iungman et al. 2023) and incorporate reflective materials to mitigate the urban heat island effect while improving ventilation corridors. Regarding the energy transition, retrofitting northern European buildings for greater efficiency and scaling up solar-powered cooling systems in southern cities will be essential to meet the evolving energy demands of the region. In the health sector, localized heat action plans, with real-time warning systems and dedicated cooling shelters, must target vulnerable populations. Moreover, in regions where outdoor labour remains a key economic activity, such as parts of eastern and southern Europe, policy interventions should enforce mandatory occupational heat safety standards, including regulated work-rest cycles, access to hydration, and shaded rest areas. Finally, research priorities include expanding UTCI monitoring to capture microclimate variations and integrating socioeconomic data for more refined vulnerability assessments, thus enabling more precise adaptation strategies.

## Conclusions

This study investigated long-term changes in thermal stress over Europe from 1940 to 2023 based on Universal Thermal Climate Index (UTCI), aiming to provide a long-range perspective on changes in thermal stress. The analyses revealed a continent-wide shift characterized by both spatial and temporal nuances. Overall, southern Europe consistently exhibits higher mean UTCI values, often exceeding 9$^{\circ }$C, indicating predominantly no to moderate thermal stress. In contrast, northern and high-altitude regions face lower values, frequently below 0$^{\circ }$C, reflecting the persistence of cold stress. At the same time, distinct regional trends emerge: while eastern European countries show the most rapid warming at over 0.3$^{\circ }$C$\cdot$decade$^{-1}$, western Europe experiences more modest trends, likely tempered by its proximity to the Atlantic Ocean.

Underlying these thermal changes are several key meteorological drivers. Air temperature and mean radiant temperature makes the strongest contributions to UTCI increases, particularly in northern and central Europe, where they have increased by 0.2–0.6$^{\circ }$C$\cdot$decade$^{-1}$. Meanwhile, relative humidity shows a notable decline, about 0.8%$\cdot$decade$^{-1}$, in Scandinavia and parts of the Mediterranean region, while reduced wind speeds in countries like Hungary and Romania amplify heat stress in continental interiors.

Evaluating annual patterns showed a gradual transition towards milder conditions: hours with no to moderate thermal stress have increased by about 0.6% $\cdot$decade$^{-1}$, reflecting fewer extreme cold events. Although heat stress starts from a smaller baseline, it has increased by approximately 0.06% $\cdot$decade$^{-1}$, especially in southern Europe. The monthly changes in thermal stress show a decline in cold stress during winter (notably January at -1.93% $\cdot$decade$^{-1}$ and February at -1.49% $\cdot$decade$^{-1}$) and pronounced heat stress increases in July and August (approximately +0.27% and +0.25% $\cdot$decade$^{-1}$, respectively).

In northern Europe, cold stress remains prevalent, with a decreasing trend of 8–12 hours $\cdot$year$^{-1}$, indicating progressively shorter winter conditions. By contrast, southern Europe shows an increase of 4–6 hours $\cdot$year$^{-1}$ in the Mediterranean region. When focusing on individual cities, northern cities like Reykjavik (Iceland) and Rovaniemi (Finland) exhibit marked reductions in cold stress, about 10 fewer hours $\cdot$year$^{-1}$, while cities in the Mediterranean basin, such as Antalya (Turkey) and Athens (Greece) showed an increase of 3–4 heat-stress hours $\cdot$year$^{-1}$. To limit the impact of Europe’s changing thermal landscape, it is necessary to focus on urban cooling solutions, improve energy resilience, and provide strong health protections.

The heat vulnerability index reveals pronounced north–south and east–west gradients: southern and eastern European regions, especially parts of Spain, Italy, Greece, Bulgaria, and Romania, face the highest heat vulnerability (HVI >0.5) due to a high number of hours with heat stress combined with older, poorer populations, whereas most of Western and Northern Europe remains relatively resilient (HVI < 0.3) due to stronger adaptive capacity.

The results of the present study represent useful benchmarks for the European policymakers, urban planners and public health officials.

## Supplementary Information

Below is the link to the electronic supplementary material.

Supplementary file 1 (docx 17873 KB)

## Data Availability

All data used in this study are publicly available through the Copernicus Climate Change Service (C3S) Climate Data Store (CDS). Specifically: “ERA5-HEAT (Human thErmAl comforT)”, Copernicus Climate Change Service (C3S) Climate Data Store (CDS), DOI: 10.24381/cds.553b7518 (Accessed on 01-Oct-2024), and “ERA5 hourly data on single levels from 1940 to present”, Copernicus Climate Change Service (C3S) Climate Data Store (CDS), DOI: 10.24381/cds.adbb2d47 (Accessed on 01-Oct-2024). These datasets are distributed under the Copernicus User License and may be accessed from C3S ClimateData Store. Where applicable, any scripts used to process or analyze these data can be provided upon request. No additional data were generated during this study. All results discussed in the paper can be reproduced using the publicly available datasets and the accompanying scripts.

## Footnotes

1 https://cran.r-project.org/web/packages/trend/index.html, accessed on 3 February 2025.

2 https://cran.r-project.org/web/packages/ClimInd/index.html, accessed on 3 February 2025.

3 https://ec.europa.eu/eurostat/web/main/data/database, accessed on 29 July 2025

## Code availability

The code used for data analysis in this study is not publicly available in a repository, but it can be obtained upon request. Researchers interested in accessing the code should contact the corresponding author.
