# Supplementary material for: Changes in the thermal stress across Europe between 1940–2023
Source: Int J Biometeorol. 2025 Oct 7;69(12):3569–86. doi: 10.1007/s00484-025-03043-x (PMC12689724; doi:10.1007/s00484-025-03043-x)
Supplement: Supplementary file 1 — (docx 17873 KB) [file 484_2025_3043_MOESM1_ESM.docx]

Supplementary Material

# Supplementary Tables

**Table S1. UTCI values (^o^C), the thermal stress categories and the recommended protection measures that need to be taken for each category [adapted from** Blazejczyk et al. (2010, 2013), DiNapoli et al. (2019, 2023)].

|  | | | |
| --- | --- | --- | --- |
| UTCI range (°C) | Stress category | Physiological responses | Protection measures |
| >46 | extreme heat stress | steep decrease in total net heat loss  averaged sweat rate >650 g h^-1^  steep increase | temporary body cooling  drinking >0.5 l h^-1^  no physical activity |
| 38 to 46 | very strong heat stress | low core to skin temperature gradient | temporary use of air conditioner  shaded places necessary  drinking >0.5 l h^-1^  reduce physical activity |
| 32 to 38 | strong heat stress | averaged sweat rate >200 g h^-1^  instantaneous change in skin temperature | use shaded places  drinking >0.25 l h^-1^  temporary reduce physical activity |
| 26 to 32 | moderate heat stress | change of slopes in sweat rate  and skin temperature  occurrence of sweating at 30 min | drinking >0.25 l h^-1^ |
| 9 to 26 | no thermal stress | averaged sweat rate >100 g h^-1^ | physiological thermoregulation  sufficient to keep thermal comfort |
| 0 to 9 | slight cold stress | local minimum of hand skin temperature | use gloves and hat |
| -13 to 0 | moderate cold stress | vasoconstriction  face skin temperature  at 30 min < 15°C (pain) | intensify activity and protect face  and extremities against cooling |
| -27 to -13 | strong cold stress | numbness  increase in core to skin  temperature gradient | intensify activity and protect face  and extremities against cooling  use warmer clothing |
| -40 to -27 | very strong cold stress | frostbite  numbness  shivering | intensify activity and protect face and extremities against cooling use warmer clothing reduce outdoor exposure time |
| < -40 | extreme cold stress | frostbite | stay at home; if outdoor  exposure is necessary, use  heavy and wind protected clothing |

**Table S2. Cities analyses in this study. For each country, three cities were selected: (i) the capital (C), (ii) the second largest city by population (SL), and (iii) the principal tourist destination (TD). For**

**Liechtenstein, Monaco, San Marino, and Vatican City only one city was selected. For each city the number of hours with heat and cold stress and the trend in the number of hours with heat and cold stress (hours yr^-1^) between 1940–2023 are indicated.**

| Country | Type | City | Heat stress mean | Heat stress trend | Cold stress mean | Cold stress trend |
| --- | --- | --- | --- | --- | --- | --- |
| Austria | C | Vienna | 92.690 | 1.177 | 947.654 | -6.864 |
| Austria | SL | Graz | 54.036 | 0.750 | 344.214 | -5.073 |
| Austria | TD | Salzburg | 8.012 | 0.074 | 516.060 | -3.337 |
| Belarus | C | Minsk | 12.142 | 0.057 | 2520.380 | -10.822 |
| Belarus | SL | Gomel | 44.214 | 0.626 | 2003.869 | -11.096 |
| Belarus | TD | Brest | 41.880 | 0.666 | 1555.690 | -8.377 |
| Belgium | C | Brussels | 37.643 | 0.313 | 560.976 | -4.045 |
| Belgium | SL | Antwerp | 34.333 | 0.333 | 473.167 | -3.877 |
| Belgium | TD | Bruges | 0.750 | 0.000 | 939.500 | -5.076 |
| Bulgaria | C | Sofia | 72.833 | 0.681 | 480.405 | -3.833 |
| Bulgaria | SL | Plovdiv | 431.333 | 1.321 | 249.476 | -2.641 |
| Bulgaria | TD | Varna | 95.810 | 0.884 | 533.488 | -3.658 |
| Croatia | C | Zagreb | 268.226 | 2.545 | 257.583 | -3.156 |
| Croatia | SL | Split | 19.714 | 0.386 | 164.631 | -0.958 |
| Croatia | TD | Dubrovnik | 253.345 | 2.545 | 164.369 | -1.373 |
| Czech Republic | C | Prague | 45.560 | 0.583 | 830.250 | -6.828 |
| Czech Republic | SL | Brno | 23.583 | 0.354 | 1096.238 | -6.600 |
| Czech Republic | TD | Karlovy Vary | 14.476 | 0.143 | 1127.548 | -7.188 |
| Denmark | C | Copenhagen | 4.702 | 0.000 | 1358.357 | -7.526 |
| Denmark | SL | Aarhus | 4.071 | 0.000 | 1141.464 | -6.798 |
| Denmark | TD | Odense | 5.643 | 0.000 | 1337.167 | -7.016 |
| Estonia | C | Tallinn | 5.560 | 0.000 | 2064.643 | -8.799 |
| Estonia | SL | Tartu | 9.417 | 0.062 | 2190.381 | -9.119 |
| Estonia | TD | Pärnu | 10.274 | 0.083 | 1781.929 | -8.976 |
| Finland | C | Helsinki | 3.893 | 0.000 | 2123.643 | -7.725 |
| Finland | SL | Espoo | 3.988 | 0.000 | 1943.464 | -7.873 |
| Finland | TD | Rovaniemi | 0.917 | 0.000 | 2819.631 | -6.040 |
| France | C | Paris | 57.929 | 0.479 | 329.393 | -3.323 |
| France | SL | Marseille | 3.274 | 0.000 | 572.464 | -3.229 |
| France | TD | Nice | 50.429 | 0.902 | 23.464 | -0.333 |
| Germany | C | Berlin | 47.893 | 0.563 | 783.440 | -6.265 |
| Germany | SL | Hamburg | 22.131 | 0.173 | 709.214 | -5.879 |
| Germany | TD | Munich | 65.440 | 0.644 | 681.143 | -6.000 |
| Greece | C | Athens | 628.369 | 1.714 | 22.333 | 0.038 |
| Greece | SL | Thessaloniki | 504.952 | 2.250 | 117.881 | -1.240 |
| Greece | TD | Heraklion | 346.298 | 1.130 | 12.202 | 0.000 |
| Hungary | C | Budapest | 123.405 | 1.215 | 606.107 | -5.000 |
| Hungary | SL | Debrecen | 224.548 | 2.024 | 628.798 | -4.811 |
| Hungary | TD | Szentendre | 123.405 | 1.215 | 606.107 | -5.000 |
| Iceland | C | Reykjavik | 0.000 | 0.000 | 2013.917 | -4.579 |
| Iceland | SL | Akureyri | 0.000 | 0.000 | 1879.393 | -9.515 |
| Iceland | TD | Vík | 0.000 | 0.000 | 3167.036 | -0.953 |
| Ireland | C | Dublin | 0.000 | 0.000 | 614.512 | -0.781 |
| Ireland | SL | Cork | 1.274 | 0.000 | 192.095 | 0.087 |
| Ireland | TD | Galway | 1.619 | 0.000 | 254.679 | 0.293 |
| Italy | C | Rome | 360.429 | 3.190 | 86.202 | -0.674 |
| Italy | SL | Milan | 454.881 | 4.252 | 103.214 | -2.000 |
| Italy | TD | Venice | 278.583 | 2.914 | 149.714 | -1.352 |
| Latvia | C | Riga | 10.119 | 0.000 | 1771.012 | -8.749 |
| Latvia | SL | Daugavpils | 13.607 | 0.096 | 1790.000 | -9.958 |
| Latvia | TD | Jurmala | 7.845 | 0.042 | 1787.679 | -8.595 |
| Liechtenstein | C | Vaduz | 29.167 | 0.448 | 356.440 | -3.400 |
| Lithuania | C | Vilnius | 12.821 | 0.100 | 1916.893 | -9.490 |
| Lithuania | SL | Kaunas | 20.869 | 0.227 | 1494.440 | -9.218 |
| Lithuania | TD | Palanga | 0.298 | 0.000 | 2480.417 | -8.410 |
| Luxembourg | C | Luxembourg City | 37.976 | 0.413 | 617.976 | -4.528 |
| Luxembourg | SL | Esch-sur-Alzette | 30.036 | 0.336 | 715.988 | -4.742 |
| Luxembourg | TD | Vianden | 28.024 | 0.283 | 675.643 | -4.640 |
| Malta | C | Valletta | 91.976 | 1.478 | 39.417 | -0.396 |
| Malta | SL | Birkirkara | 91.976 | 1.478 | 39.417 | -0.396 |
| Malta | TD | Mdina | 91.976 | 1.478 | 39.417 | -0.396 |
| Moldova | C | Chișinău | 155.262 | 1.496 | 828.476 | -5.936 |
| Moldova | SL | Bălți | 160.667 | 1.682 | 817.131 | -6.037 |
| Moldova | TD | Orhei | 154.810 | 1.571 | 832.024 | -6.000 |
| Monaco | C | Monaco | 50.429 | 0.902 | 23.464 | -0.333 |
| Montenegro | C | Podgorica | 113.738 | 1.316 | 262.095 | -2.452 |
| Montenegro | SL | Nikšić | 19.012 | 0.176 | 557.726 | -2.466 |
| Montenegro | TD | Kotor | 112.464 | 1.545 | 200.607 | -1.763 |
| Netherlands | C | Amsterdam | 22.405 | 0.201 | 594.643 | -4.741 |
| Netherlands | SL | Rotterdam | 15.464 | 0.128 | 749.167 | -5.107 |
| Netherlands | TD | The Hague | 0.798 | 0.000 | 1060.571 | -5.877 |
| North Macedonia | C | Skopje | 296.214 | 2.549 | 194.405 | -2.186 |
| North Macedonia | SL | Bitola | 23.667 | 0.129 | 191.405 | -1.239 |
| North Macedonia | TD | Ohrid | 8.774 | 0.000 | 169.429 | -0.957 |
| Norway | C | Oslo | 2.607 | 0.000 | 1355.095 | -8.345 |
| Norway | SL | Bergen | 0.000 | 0.000 | 1755.929 | -3.912 |
| Norway | TD | Tromsø | 0.774 | 0.000 | 2737.405 | -9.596 |
| Poland | C | Warsaw | 46.429 | 0.561 | 1178.452 | -7.406 |
| Poland | SL | Kraków | 42.286 | 0.571 | 937.988 | -7.495 |
| Poland | TD | Gdańsk | 11.119 | 0.061 | 1737.440 | -8.388 |
| Portugal | C | Lisbon | 2.405 | 0.000 | 9.905 | -0.043 |
| Portugal | SL | Porto | 15.024 | 0.067 | 16.869 | -0.144 |
| Portugal | TD | Faro | 0.214 | 0.000 | 9.667 | -0.059 |
| Romania | C | Bucharest | 298.905 | 1.570 | 627.833 | -4.230 |
| Romania | SL | Cluj-Napoca | 31.131 | 0.361 | 678.155 | -4.952 |
| Romania | TD | Brașov | 16.905 | 0.193 | 665.119 | -4.435 |
| Russia  (Western part) | C | Moscow | 26.714 | 0.143 | 2408.262 | -8.495 |
| Russia  (Western part) | SL | Saint Petersburg | 7.036 | 0.000 | 2495.488 | -8.054 |
| Russia  (Western part) | TD | Sochi | 96.440 | 1.671 | 128.857 | -0.520 |
| San Marino | C | San Marino | 335.262 | 2.842 | 103.726 | -0.982 |
| Serbia | C | Belgrade | 316.429 | 2.018 | 516.000 | -4.447 |
| Serbia | SL | Novi Sad | 331.429 | 2.000 | 484.881 | -4.255 |
| Serbia | TD | Niš | 280.417 | 1.890 | 245.155 | -2.946 |
| Slovakia | C | Bratislava | 124.429 | 1.304 | 869.214 | -6.320 |
| Slovakia | SL | Košice | 27.440 | 0.333 | 779.786 | -6.113 |
| Slovakia | TD | Piešťany | 56.750 | 0.794 | 783.226 | -5.688 |
| Slovenia | C | Ljubljana | 46.786 | 0.750 | 406.143 | -4.257 |
| Slovenia | SL | Maribor | 104.143 | 1.295 | 363.024 | -4.346 |
| Slovenia | TD | Piran | 30.714 | 0.741 | 278.167 | -1.956 |
| Spain | C | Madrid | 254.893 | 2.190 | 124.595 | -1.321 |
| Spain | SL | Barcelona | 65.048 | 1.000 | 20.595 | -0.264 |
| Spain | TD | Seville | 970.774 | 1.652 | 5.119 | 0.000 |
| Sweden | C | Stockholm | 1.286 | 0.000 | 1661.405 | -7.040 |
| Sweden | SL | Gothenburg | 0.179 | 0.000 | 1587.833 | -7.433 |
| Sweden | TD | Visby | 0.131 | 0.000 | 2026.857 | -6.826 |
| Switzerland | C | Bern | 45.810 | 0.600 | 299.345 | -4.887 |
| Switzerland | SL | Zürich | 67.929 | 0.828 | 432.750 | -5.000 |
| Switzerland | TD | Lucerne | 28.452 | 0.392 | 222.202 | -3.828 |
| Turkey  (Western part) | C | Ankara | 308.143 | 2.460 | 334.333 | -3.847 |
| Turkey  (Western part) | SL | Istanbul | 6.631 | 0.048 | 506.821 | -2.682 |
| Turkey  (Western part) | TD | Antalya | 953.012 | 1.422 | 71.881 | -0.250 |
| United Kingdom | C | London | 9.142 | 0.000 | 411.107 | -2.453 |
| United Kingdom | SL | Birmingham | 5.154 | 0.000 | 456.964 | -2.469 |
| United Kingdom | TD | Edinburgh | 0.261 | 0.000 | 703.452 | -1.706 |
| Ukraine | C | Kyiv | 61.381 | 0.762 | 1494.131 | -8.409 |
| Ukraine | SL | Kharkiv | 116.167 | 1.105 | 1644.655 | -8.307 |
| Ukraine | TD | Lviv | 37.786 | 0.467 | 1531.905 | -8.601 |
| Vatican City | C | Vatican City | 360.429 | 3.190 | 86.202 | -0.674 |

1. Supplementary figures

**Figure S1** – The location the 118 cities anlyzed in the study.

**Figure S2** - Spatial distribution of (a)–(b) the annual mean and (c) – (d) trend between 1940–2023 for 2 m air temperature (a) and (c) and relative humidity (b) and (d). Dots in the panel (c) and (d) are indicating statistically significant monotonic trends (p < 0.05).

**Figure S3** - As in Fig. S1 but for wind speed (a) and (c), and mean radiant temperature (b) and (d).

**Figure S4** - The spatial distribution of the interaction term (ΔUTCI_interaction_). See section 2.2 for details on how this term is defined in “one-at-a-time” perturbation approach.
